# Supplementary material for: Segmenting functional tissue units across human organs using community-driven development of generalizable machine learning algorithms
Source: bioRxiv. 2023 Jan 6:2023.01.05.522764. Preprint. [Version 1] doi: 10.1101/2023.01.05.522764 (PMC9881902; doi:10.1101/2023.01.05.522764)
Supplement: 1 [file NIHPP2023.01.05.522764V1-supplement-1.pdf]

bioRxiv preprint doi: https://doi.org/10.1101/2023.01.05.522764; this version posted January 6, 2023. The copyright holder for this preprint (which was not certified by peer review) is the author/funder, who has granted bioRxiv a license to display the preprint in perpetuity. It is made available under aCC-BY 4.0 International license.

Supplementary Information

Supplementary Figures

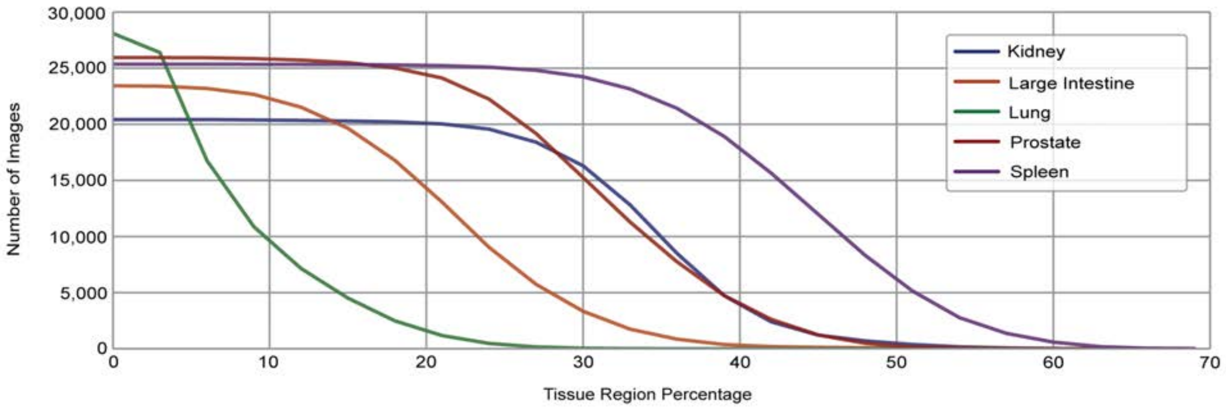

Supplementary Figure 1. Tissue region percentage vs. number of images against different threshold values for public HPA data for all five organs. Final selected tissue region percentage thresholds: 5% for lung and 15% for other four organs.

bioRxiv preprint doi: https://doi.org/10.1101/2023.01.05.522764; this version posted January 6, 2023. The copyright holder for this preprint (which was not certified by peer review) is the author/funder, who has granted bioRxiv a license to display the preprint in perpetuity. It is made available under aCC-BY 4.0 International license.

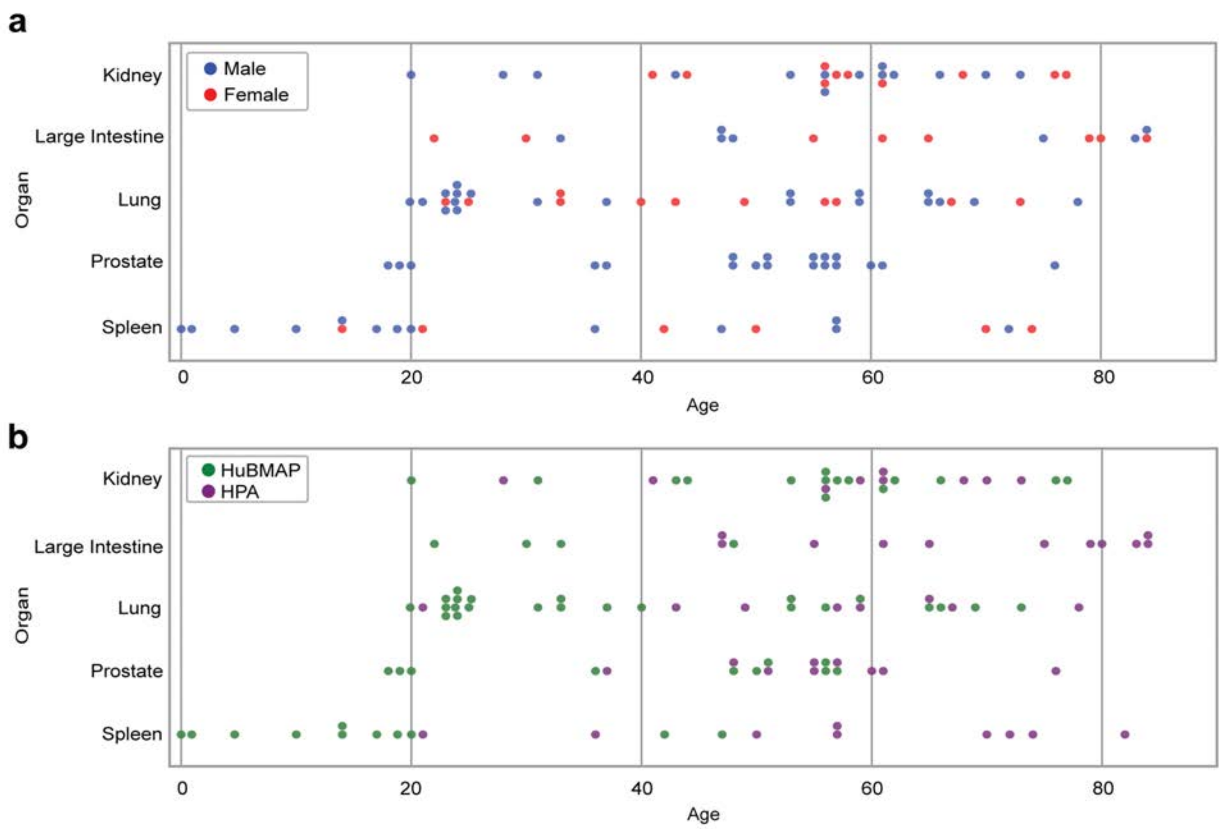

Supplementary Figure 2. Tissue samples per organ and age for all 5 organs. a. Donor distribution color coded by male (blue) and female (red). **b.** Donor age and sex distribution color coded by HPA (purple) and HuBMAP (green).

bioRxiv preprint doi: https://doi.org/10.1101/2023.01.05.522764; this version posted January 6, 2023. The copyright holder for this preprint (which was not certified by peer review) is the author/funder, who has granted bioRxiv a license to display the preprint in perpetuity. It is made available under aCC-BY 4.0 International license.

Supplementary Notes

Supplementary Notes 1. Judges' Prizes Rubric

1.1 Scientific Prize

Kaggle teams were asked to investigate correlations between predicted FTUs (e.g., area and shape) and donor demographics (e.g., sex and age). The evaluation rubric further emphasized validation of methods and implementations, documentation of performance and limitations, novelty of solutions, and presentation of insights useful for generation of reference FTUs for inclusion into a Human Reference Atlas. Scientific Prize winners were identified by a panel of human experts who selected two teams to receive equal Scientific Prize amounts ($10,000 each) based on the submission's contribution to science and demonstration of innovative approaches. The complete evaluation rubric, as presented below, consisted of eight criteria which were used by the judges to evaluate the winners. Each criterion consisted of ten points for a total of 80 points.

1. Are the statistical and modeling methods used to identify FTUs appropriate for the task?
2. Are confidence scores and other metrics provided that help interpret the results achieved by the segmentation methods?
3. Is the presented characterization of FTUs useful for understanding individual differences, e.g., the impact of donor sex and age on the shape, size or spatial distribution of FTUs?

bioRxiv preprint doi: https://doi.org/10.1101/2023.01.05.522764; this version posted January 6, 2023. The copyright holder for this preprint (which was not certified by peer review) is the author/funder, who has granted bioRxiv a license to display the preprint in perpetuity. It is made available under aCC-BY 4.0 International license.

4. Is it possible to predict FTU area size distribution, given age and sex information across all organs?
5. Did the team validate their methods and algorithm implementations and provide information on algorithm performance and limitations?
6. Did the team document their method and code appropriately?
7. Did the team develop a creative or novel method to segment FTUs?
8. Did the team provide insights that would be useful for generating reference FTUs for inclusion into a HUman Reference Atlas?

1.2 Diversity and Presentation Prize

The complete evaluation rubric, as presented below, consisted of three criteria which were used by the judges to evaluate the winner. Each criterion consisted of ten points for a total of 30 points.

1. Does the team embrace diversity and equity, welcoming team members of different ages, genders, ethnicities, and with multiple backgrounds and perspectives?
2. Did the authors effectively communicate the details of their method for segmenting FTUs, and the quality and limitations of their results? For example, did they use data visualizations to present algorithm setup, run, results and/or to provide insight into the comparative performance of different methods? Were these visualizations effective at communicating insights about their approach and results to experts and novice users?
3. Are the important results easily understood by the average person?

bioRxiv preprint doi: https://doi.org/10.1101/2023.01.05.522764; this version posted January 6, 2023. The copyright holder for this preprint (which was not certified by peer review) is the author/funder, who has granted bioRxiv a license to display the preprint in perpetuity. It is made available under aCC-BY 4.0 International license.

Supplementary Notes 2. Alveoli segmentations in lung tissue

Due to confusion regarding varied looking alveoli segmentations in lung tissue images, additional information was provided to the teams. The data included masks of both atelectatic (collapsed) and inflated alveoli (un-collapsed). The alveolar appearance on the image slides depends on how the tissue samples were prepared. For the inflated alveoli, which have a 3D ‘cup’ shaped structure, how the tissue is sectioned can cause variability as well. If the alveoli were sectioned in a horizontal manner, their shape will appear more like a complete circle. Whereas if the alveoli were sectioned vertically, they may appear more as a U-shape.

bioRxiv preprint doi: https://doi.org/10.1101/2023.01.05.522764; this version posted January 6, 2023. The copyright holder for this preprint (which was not certified by peer review) is the author/funder, who has granted bioRxiv a license to display the preprint in perpetuity. It is made available under aCC-BY 4.0 International license.

Supplementary Table Legends

All tables can be accessed at Supplementary Tables.

Supplementary Table 1. Team 1 Ablation Study. This table lists the ablation study done by the winning team, detailing the strategies that helped improve the performance of their solution.

SUPPLEMENTARY TABLE 1: Team 1 Ablation Study

Model	Private Dice	Public Dice	Public Hubmap Dice	Private Dice Gain
mit-b4 + lung annotation	0.79318		0.58097	0.07256
mit-b4 + prostate_downscale	0.68486		0.52633	0.05791
mit-b4 + albu dataaug + pseudo label(prostate, intestine)	0.72062	0.76051	0.54628	0.02496
mit-b4 + better resize	0.69566		0.52913	0.0108
mit-b4 + stain transfer(torchstain)	0.80838	0.80858	0.59243	0.00927
mit-b4 aug + brightness	0.82451		0.6046	0.00853
mit-b4 + external lung	0.82054	0.81345	0.60057	0.00622
mit-b4 + external spleen	0.81432		0.59722	0.00594
mit-b4 + trainval(351 images)	0.79799	0.80006	0.58248	0.00481
3 model ensemble	0.83238	0.82364		0.00417
mit-b4_optimized_threshold	0.82786		0.60663	0.00335
4 model ensemble + image ration divisor 32	0.8338	0.82622		0.00142
mit-b4 + pseudo label(prostate, intestine) patch	0.79911		0.58466	0.00112
6 model ensemble	0.83562	0.82716		0.00094
5 model ensemble	0.83468	0.82679		0.00088
mit-b4_0.8_1_1.2	0.82821		0.60718	0.00035
mit-b4	0.62695		0.47984	0
mit-b4 aug + HPA original (not stained)	0.81598	0.81661		-0.00456

* Sorted in increasing order of private gain.

** List is non-exhaustive and does not include all experiments by the team. Only listed is the subset of experiments team tracked and provided.

bioRxiv preprint doi: https://doi.org/10.1101/2023.01.05.522764; this version posted January 6, 2023. The copyright holder for this preprint (which was not certified by peer review) is the author/funder, who has granted bioRxiv a license to display the preprint in perpetuity. It is made available under aCC-BY 4.0 International license.

Supplementary Table 2: Team 3 Ablation Study. This table lists the ablation study done by the team that won the third performance prize, detailing the strategies that helped improve the performance of their solution.

SUPPLEMENTARY TABLE 2: Team 3 Ablation Study

Model	Out of Fold Dice	Public HuBMAP Dice	Private Dice	Private Dice Gain
Baseline	0.71172	0.12115	0.15632	-
+ Pixel size adaptation	0.71588	0.22653	0.30195	0.14563
+ Histogram matching	0.70283	0.38732	0.48486	0.18291
+ 1 output channel + CutMix	0.75157	0.49483	0.64518	0.16032
+ Heavy augmentations	0.7695	0.51506	0.71187	0.06669
+ additional scalers + external GTEX data with pseudo labels	0.82142	0.58037	0.78352	0.07165
+ additional HPA data with pseudo labels	0.83633	0.598	0.81402	0.0305
+ Best 5 folds solo model	0.85405	0.60826	0.83332	0.0193
+ Ensemble	0.85428	0.60931	0.83419	0.00087

* Public HuBMAP Dice involves setting predictions for public test HPA data to zero.

** List is non-exhaustive and does not include all experiments by the team. Only listed is the subset of experiments team tracked and provided.

bioRxiv preprint doi: https://doi.org/10.1101/2023.01.05.522764; this version posted January 6, 2023. The copyright holder for this preprint (which was not certified by peer review) is the author/funder, who has granted bioRxiv a license to display the preprint in perpetuity. It is made available under aCC-BY 4.0 International license.

Supplementary Table 3: Organ Dice Score Comparison for Teams Winning the Performance Prizes. This table provides the organ-wise breakdown of performance results for the three winning teams.

Supplementary Table 3: Organ Dice Score Comparison for 3 Winning Teams

Team	Kidney	Large Intestine	Lung	Prostate	Spleen	Overall
First place winner	0.96401	0.89676	0.72664	0.85004	0.83862	0.83562
Second place winner	0.9665	0.88931	0.72092	0.84851	0.84157	0.83393
Third place winner	0.9491	0.86232	0.73599	0.84806	0.84211	0.83266

* All scores presented for private test set.
